# Our Janitor

**Published:** 1858-05

**Authors:** 


Our Janitor, Hans Anderson, has on hand several carefully prepared Skeletons, which he will sell at very low prices. He may be addressed upon the subject through Box 2802, Chicago Post Office.

